# Enhanced Adsorption of Bisphenol A from Aqueous Solution with 2-Vinylpyridine Functionalized Magnetic Nanoparticles

**DOI:** 10.3390/polym10101136

**Published:** 2018-10-12

**Authors:** Qiang Li, Fei Pan, Wentao Li, Dongya Li, Haiming Xu, Dongsheng Xia, Aimin Li

**Affiliations:** 1 School of Environmental Engineering, Wuhan Textile University, Wuhan 430073, China; lqspecial@163.com (Q.L.); leopan0724@189.cn (F.P.); dyli@wtu.edu.cn (D.L.); xulianhe@163.com (H.X.); 2 Engineering Research Center for Clean Production of Textile Dyeing and Printing, Ministry of Education, Wuhan 430073, China; 3 State Key Laboratory of Pollution Control and Resources Reuse, School of the Environment, Nanjing University, Nanjing 210023, China; liwentaonju2007@hotmail.com

**Keywords:** 2-vinylpyridine, magnetic nanoparticles, bisphenol A, adsorption, reusability

## Abstract

In this study, a novel 2-vinylpyridine functionalized magnetic nanoparticle (Mag-PVP) was successfully prepared. The prepared Mag-PVP was characterized by transmission electronic microscopy (TEM), Fourier transform infrared spectrophotometry (FT-IR), vibrating sample magnetometry (VSM) and thermogravimetric analysis (TGA), and was used for the adsorption of bisphenol A (BPA) from aqueous solutions. Mag-PVP, which is composed of Fe_3_O_4_ nanoparticles and poly divinylbenzene-2-vinylpyridine (with a thickness of 10 nm), exhibited magnetic properties (*M*_s_ = 44.6 emu/g) and thermal stability. The maximum adsorption capacity (*Q_m_*) of Mag-PVP for BPA obtained from the Langmuir isotherm was 115.87 mg/g at 20 °C, which was more than that of Fe_3_O_4_ nanospheres. In the presence of NaCl, the improved adsorption capacity of Mag-PVP was probably attributed to the screening effect of Mag-PVP surface charge and salting-out effect. In the presence of CaCl_2_ and humic acid (HA), the adsorption capacity of BPA decreased due to competitive adsorption. The adsorption of BPA by Mag-PVP increased slightly with the increase in pH from 3.0 to 5.0 and obtained the largest adsorption amount at pH 5.0, which was probably attributed to hydrogen bonding interactions. Moreover, in actual water, Mag-PVP still showed excellent adsorption performance in removing BPA. The high adsorption capacity and excellent reusability performance in this work indicated that Mag-PVP was an effective adsorbent for removing BPA from aqueous solutions.

## 1. Introduction

In recent years, researchers have shown concern about the fate, transport, reactivity and bioavailability of organic micro-pollutants (OMPs) [1,2]. Among these OMPs, endocrine disrupting chemicals (EDCs) can imitate the biological activity of natural hormones, occupy the hormone receptors, or interfere with the body’s hormonal system, which can pose health risks to animals and humans [3]. Bisphenol A (2,2-bis(4-hydroxyphenyl) propane; BPA), one of these EDCs, is widely used as an important monomer for the production of plastics [4]. It is also extensively used in adhesives, plasticizers and polymerization inhibitors. The global demand for BPA is growing fast. The US market was growing at about 4.2% yearly up to 2010, with the growth of polycarbonates and epoxy resins. In Europe, the demand is expected to remain steady while the strongest growth will be in China [5]. The widely used BPA products lead to a gradual release of BPA into the environment, where it is frequently detected in industrial wastewater, groundwater, surface water, and even drinking water [6,7]. Therefore, efficient treatment processes have to be developed for the removal of BPA.

Currently, various technologies are being used to remove BPA from water, including adsorption [8,9], membrane separation [10], biological treatment [11], oxidation degradation [4], and other processes. Among these methods, adsorption has been found to be superior to other techniques for BPA removal based on low cost, flexibility, and fewer harmful secondary products [12]. A large number of adsorbents have been studied for the removal of BPA, such as active carbon [8], graphene [13], carbon nanotubes (CNTs) [14], resin polymers [15], mineral materials [16], etc. However, the use of these adsorbents on a large scale is limited by the difficulties of separation and regeneration. In order to solve the difficulty in collecting adsorbents from their dispersing media, much attention has been paid to the combination of magnetic nanoparticles and conventional adsorbents. Li et al. [17] prepared a recyclable magnetic carbon nanotube (CNTs/Fe_3_O_4_) for removing BPA from water. Pan et al. [18] synthesized the chitosan/fly-ash-cenospheres/γ-Fe_2_O_3_ (CTS/γ-Fe_2_O_3_/FACs) magnetic composites for the removal of BPA from aqueous solutions. Yantasee et al. [19] utilized thiol functionalized superparamagnetic nanoparticles to remove heavy metals from aqueous systems. Polydivinylbenzene magnetic latex particles were prepared for the removal of BPA and some heavy metal ions [20]. Moreover, Zhang et al. [21] found that magnetite nanoparticles (Fe_3_O_4_ MNPs) exhibited ultrahigh adsorption towards chlorotetracycline, and many other studies suggested that the magnetic composite adsorbent could remove pollutants [22,23,24]. These adsorbents not only possessed the adsorption advantages of the monomer, but also could be easily separated from the solutions by an external magnet. Research indicated that the 2-vinylpyridine (2-VP) polymer had a high adsorption capacity towards aromatic acids and metal ions [25,26]; 2-VP is also a good hydrogen bonding acceptor [27]. The composite adsorbent of 2-VP and Fe_3_O_4_ may have better adsorption performance. However, to our knowledge, no relevant work regarding the application of a 2-VP and Fe_3_O_4_ composite adsorbent for BPA removal has been reported.

The objective of the present study was to study: (1) the preparation and physicochemical properties of a composite adsorbent of 2-VP and Fe_3_O_4_ (Mag-PVP); (2) the adsorption behavior of Mag-PVP for the removal of BPA from aqueous solution, analyzing the influence of the chemistry of solution (ionic strength, humic acid, and pH) on this process; (3) the adsorption characteristic and mechanism. 

## 2. Experimental Section

### 2.1. Chemicals

All chemicals were of analytical reagent grade. The polymer 2-vinylpyridine (2-VP) was purchased from Shandong LuKe Chemical Co., Ltd. (Qingdao, China). Divinylbenzene (DVB, 80%, wt %, J&K Scientific Ltd., Beijing, China) was washed with 20% (wt %) sodium hydroxide solution and dried with anhydrous sodium sulfate prior to its use. Ferric chloride (FeCl_3_·6H_2_O), ethylene glycol, sodium acetate (NaAc), γ-methacryloxypropyltrimethoxysilane (γ-MPS, 98%), ammonium solution (25% *w*/*w*), alcohol, methanol, 2, 2′-azobisisobutyronitrile (AIBN), HCl, NaOH, NaCl, CaCl_2_, and bisphenol A (BPA) were supplied from Nanjing Chemical Reagent Company (Nanjing, China). Humic acid (HA) was purchased from J&K Chemical, Ltd. (Beijing, China). The stock solution for BPA (50 mg/L) was prepared by dissolving a suitable amount of solid reagent with deionized water. The Amberlite XAD-4 was purchased from Rohm & Haas Company (Philadelphia, PA, USA).

### 2.2. Syntheses of Fe_3_O_4_ Nanospheres

Fe_3_O_4_ nanospheres were prepared through a solvothermal reaction according to a previous report [28]. FeCl_3_·6H_2_O (33.75 g) and NaAc (90 g) were added to 1 L of ethylene glycol. The mixture was stirred for 4 h to ensure all materials dissolved completely. Then, the mixture was transferred to a Teflon-lined stainless-steel autoclave and sealed for heating at 180 °C for 8 h. The precipitated black products were collected with an external magnet and washed with ethanol six times. 

### 2.3. Preparation of γ-MPS-Modified Fe_3_O_4_ Nanospheres

After washing with ethanol, the magnetic particles were dispersed in 400 mL of ethanol, 200 mL of γ-MPS and 50 mL of ammonium solution. The mixture was stirred for 10 h under a nitrogen atmosphere at 40 °C. Then, the particles were separated by an external magnet and washed with ethanol six times.

### 2.4. Preparation of Fe_3_O_4_ Nanospheres Coated with 2-VP

After washing with ethanol, γ-MPS-modified Fe_3_O_4_ nanospheres (γ-MPS-Fe_3_O_4_) were dispersed in 200 mL of ethanol. Then, a mixture solution containing 10 g of 2-VP and 5 g of DVB was added. The mixture was stirred at 75 °C for 10 h with 0.1 g of AIBN as the initiator. After polymerization, Fe_3_O_4_ nanospheres coated with poly-divinylbenzene-2-vinylpyridine (Mag-PVP) were washed with ethanol six times to remove the unpolymerized monomer. After washing with distilled water three times, Mag-PVP nanospheres were dried at 60 °C under a nitrogen atmosphere.

### 2.5. Material Characterization

The surface morphology of the polymer was observed using a transmission electronic microscope (TEM, S-3400N II, Hitachi, Tokyo, Japan). FTIR spectrophotometry was performed with a Fourier transform infrared spectrophotometer (FTIR, Nexus870, Nicolet, Madison, WI, USA). The thermal stability of the polymer was measured using thermogravimetric analysis (Pyris 1 TGA, PerkinElmer, Norwalk, CT, USA) at a heating rate of 10 °C/min under a nitrogen atmosphere. The magnetization curves of the polymers were measured with a vibrating sample magnetometer (VSM, Quantum Design MPMS-5S, Inc., San Diego, CA, USA). The zeta potential of Mag-PVP was varied by mixing 0.1 g of the adsorbent with 200 mL of deionized water. The suspensions were stirred for 2 h to reach equilibrium potential. Following this, the solution pH was adjusted to 3–10 with 0.1 M HCl and 0.1 M NaOH. Samples (3 mL) were collected with glass syringes and analyzed by a Zeta-plus 4 instrument (Brookhaven Instrument Co., Brookhaven, TN, USA).

### 2.6. Batch Adsorption

Batch experiments were carried out in a 250 mL flask containing 50 mL of aqueous solution. The flasks were shaken at 150 rpm at 20 °C in a constant temperature vibrator (DQHZ-2001A, Taicang Huamei Instrument Co., Ltd., Taicang, China). For the adsorption equilibrium study, 0.01 g of Mag-PVP and Fe_3_O_4_ were introduced into a series of 250 mL glass conical flasks containing 50 mL of BPA solutions at different initial concentrations (2.5–50 mg/L). For the kinetic study, 0.02 g of Mag-PVP was added to 100 mL of BPA solution at a concentration of 10 mg/L. Samples were taken at 3, 5, 10, 30, 60, 90 and 120 min for the analysis of residual BPA, respectively. The effect of pH was determined by adding HCl and NaOH into 100 mL of BPA solution at a concentration of 20 mg/L. The influences of ionic strength and coexisting cations were tested by adding NaCl and CaCl_2_, respectively. The effect of HA was investigated by changing the concentration of HA from 5 to 60 mg/L, with the initial concentration of BPA fixed at 10 mg/L. The adsorption of BPA on Mag-PVP in several environmental water samples was also tested by fixing the initial concentration of BPA at 10 mg/L. All batch experiments were conducted within 4 h to reach adsorption equilibrium, except for the kinetic study. The regeneration of Mag-PVP was carried out by desorbing BPA from Mag-PVP with methanol, ethanol, toluene and cyclohexane. After reaching the adsorption equilibrium, 20 mL of methanol was added and shaken for 4 h. The regenerated resin continued to absorb BPA again, and the adsorption capacity was calculated after reaching equilibrium. All experiments were duplicated and repeated three times, and the average standard deviation of the duplicated experiments was ±10.0% for the individual composition of the reaction system.

### 2.7. HPLC Analysis

All samples were analyzed by high-performance liquid chromatography (HPLC, Agilent 1200, Waldbronn, Germany) with a diode array detector (DAD) and a reverse-phase Krornasil-C18 column (250 mm × 4.6 mm × 5 μm). The mobile phase was 1.0 mL/min of 70% methanol and 30% deionized water. The DAD detection wavelength was 221 nm and the column temperature was set at 30 °C. All samples were filtered through 0.45 μm membrane filters before detection. 

The adsorbed concentration at the equilibrium of BPA (*Q_e_*, mg/g) was calculated by:

*Q_e_* = *V* (*C*_0_ − *C_e_*)/*W* (1)

where *V* (L) is the volume of solution and *W* (g) is the weight of the dry polymer. *C*_0_ and *C_e_* represent the initial concentration and equilibrium concentration, respectively.

In the adsorption kinetic experiments, the amount of adsorbed BPA (*Q_t_*, mg/g) at a time *t* was calculated by:

*Q_t_* = *V* (*C*_0_ − *C_t_*)/*W* (2)

where *C_t_* represents the concentration at time *t* (min).

## 3. Results and Discussion

### 3.1. Characterization

**Scheme 1:**
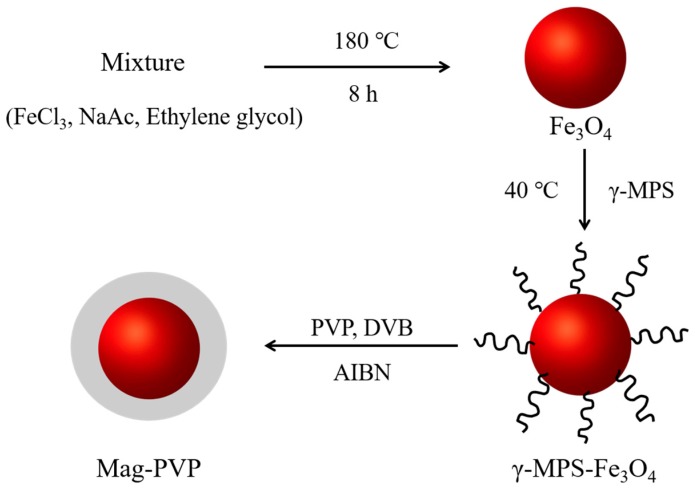
Preparation route of functionalized magnetic nanoparticles (Mag-PVP). γ-MPS = γ-methacryloxypropyltrimethoxysilane. DVB = divinylbenzene. AIBN = 2,2′-azobisisobutyronitrile. PVP = poly-divinylbenzene-2-vinylpyridine.

**Figure 1:**
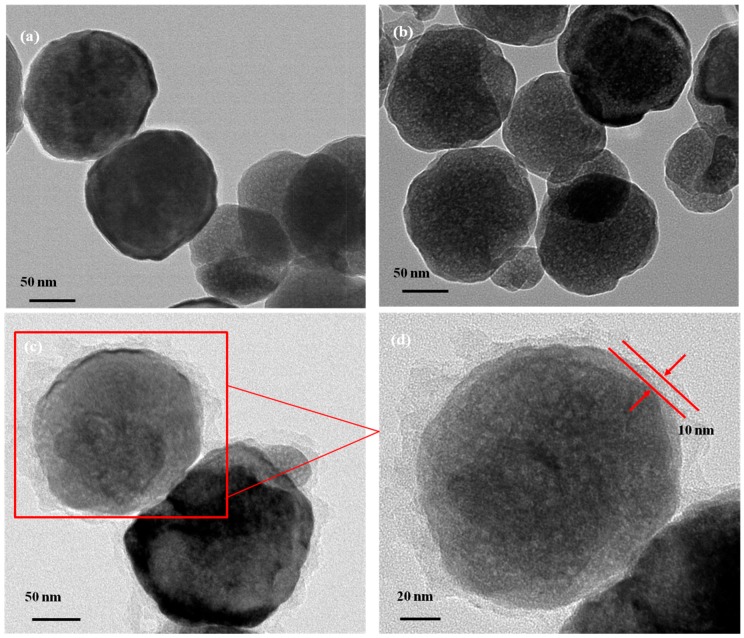
Transmission electron microscope (TEM) image of (**a**) Fe_3_O_4_, (**b**) γ-MPS-Fe_3_O_4_, and (**c**,**d**) Mag-PVP.

The Fe_3_O_4_ nanospheres were prepared through solvothermal reaction. The functionalized magnetic nanoparticles (Mag-PVP) were prepared with Fe_3_O_4_ nanospheres and PVP. Its preparation route has been diagrammatically shown in Scheme 1. The TEM images of Fe_3_O_4_, γ-MPS-Fe_3_O_4_, and Mag-PVP are presented in Figure 1a–d, respectively. The prepared magnetic nanoparticles exhibited a uniform distribution and the diameter of these nanospheres was approximately 150 nm. TEM images (Figure 1c,d) showed that the Fe_3_O_4_ nanoparticles were coated by a gray polymer shell. Based on the TEM images of Mag-PVP, the thickness of the gray polymer shell was about 10 nm. The diameter of Mag-PVP was approximately 160 nm. Details about the structure of the gray polymer shell were investigated in the following characterizations.

**Figure 2:**
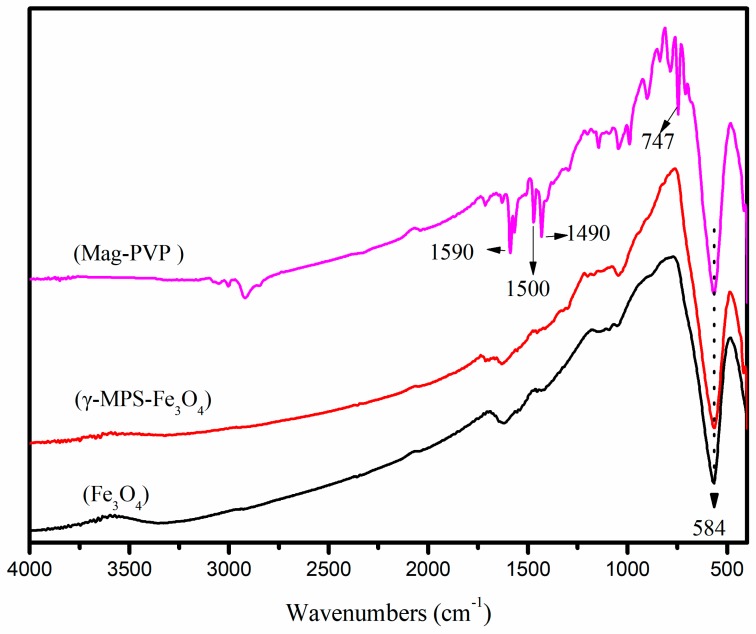
FTIR spectrum of Fe_3_O_4_, γ-MPS-Fe_3_O_4_, and Mag-PVP.

The FTIR spectra of Fe_3_O_4_, γ-MPS-Fe_3_O_4_ and Mag-PVP are shown in Figure 2. The absorption bands around 1590, 1500 and 1490 cm^−1^ of Mag-PVP were assigned to the characteristic vibration of pyridine rings, indicating the presence of PVP in the Mag-PVP [29]. The band at 747 cm^−1^ was assigned to C–H stretching. The band at 584 cm^−1^ was assigned to Fe–O stretching and bending modes [30], confirming the presence of Fe_3_O_4_ in the Mag-PVP. All these indicated that the Mag-PVP composites were successfully coated with poly-divinylbenzene-2-vinylpyridine. 

**Figure 3:**
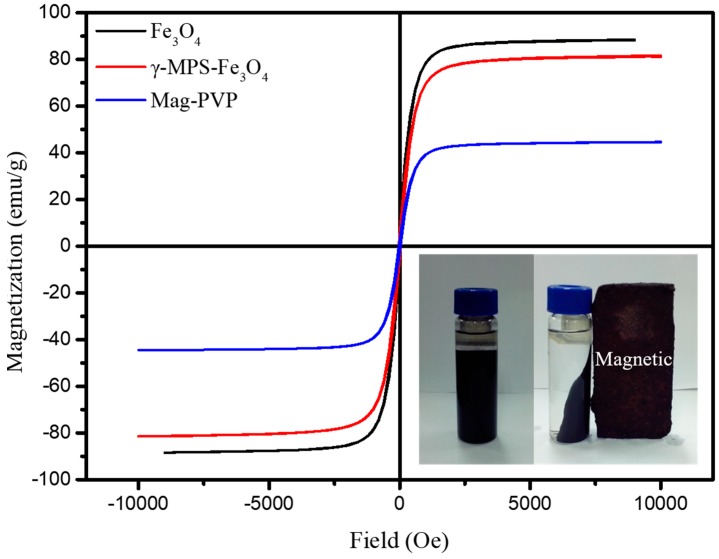
Magnetic hysteresis curves of Fe_3_O_4_, γ-MPS-Fe_3_O_4_, and Mag-PVP.

**Figure 4:**
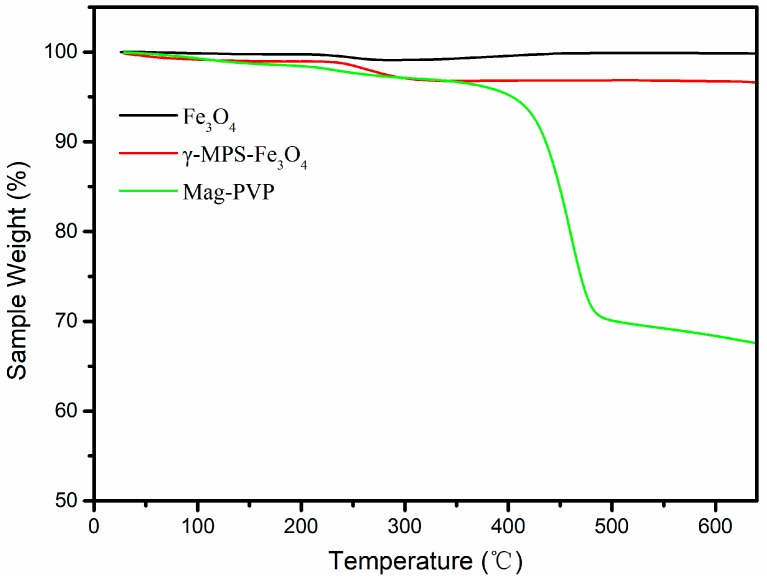
Thermogravimetric analysis (TGA) of Fe_3_O_4_, γ-MPS-Fe_3_O_4_, and Mag-PVP.

As shown in Figure 3, the magnetic hysteresis curves of the Fe_3_O_4_ and γ-MPS-Fe_3_O_4_ microspheres were 88.3 and 81.1 emu/g, respectively, which were far more than that of Mag-PVP microspheres (44.6 emu/g). The decrease in the saturation magnetization was caused by the coating layers of Fe_3_O_4_, resulting in an increase of surface disorientation [31]. The results also strongly suggested that the remaining magnetic force in Mag-PVP could be effectively attracted by an external magnetic field. Mag-PVP was a feasible magnetic separation carrier with a good magnetic characteristic. When the external magnetic field was applied, Mag-PVP microspheres were quickly separated from the solution. Figure 4 showed the thermogravimetric analysis (TGA) curves of Fe_3_O_4_, γ-MPS-Fe_3_O_4_ and Mag-PVP. In these curves, different reaction stages were observed. For Fe_3_O_4_, γ-MPS-Fe_3_O_4_ and Mag-PVP, the exponential weight decay until 250 °C was attributed to absorbed humidity or surface hydroxyls. While the weight loss of Mag-PVP in the 380–480 °C region was thought to be due to degradation of the DVB chain, this loss did not occur in the Fe_3_O_4_ and γ-MPS-Fe_3_O_4_ samples [32]. The 480–600 °C region was mainly attributed to thermo-oxidative degradation of the PVP polymer chain [33]. All of these results indicated that the thermo-stable Mag-PVP was a polymer based on magnetic nanoparticles.

### 3.2. Adsorption Equilibrium

**Figure 5:**
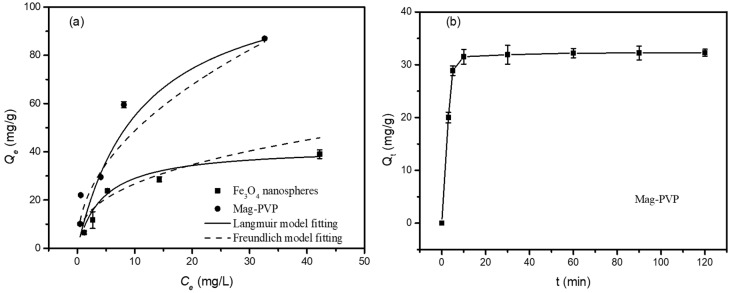
(**a**) Adsorption equilibrium for bisphenol A (BPA) on Fe_3_O_4_ nanospheres and Mag-PVP and their model fitting by Langmuir and Freundlich isotherm models (adsorbent dosage = 0.2 g/L; agitation speed = 150 rpm; shaking time = 4 h; temperature = 20 °C); (**b**) The effect of contact time on the adsorption of BPA by Mag-PVP (adsorbent dosage = 0.2 g/L; agitation speed = 150 rpm; temperature = 20 °C; BPA concentration = 10 mg/L). Error bars represent standard deviation values from three replicates. *Q_e_* = equilibrium adsorption capacity. *C_e_* = equilibrium concentration of BPA.

Figure 5 showed the different adsorption performance of BPA onto Mag-PVP and Fe_3_O_4_ nanospheres. The adsorption capacity of BPA increased with an increase in the concentration of BPA 2.5 to 50 mg/L (Figure 5a). The *Q_e_* of Mag-PVP reached 87 mg/g in BPA solution with a concentration of 50 mg/L, which was more than that of the Fe_3_O_4_ nanospheres (39 mg/g). Adsorption equilibrium data were modeled by the Langmuir [34] and Freundlich [35] isotherm models, as depicted by Equations (3) and (4), respectively:

*Q_e_* = *Q_m_K_L_C_e_*/(1 + *K_L_C_e_*) (3)

(4) $$Q_{e}=K_{F}C_{e}^{l/n}$$

where *Q_e_* (mg/g) was the equilibrium adsorption capacity and *Q_m_* (mg/g) was the maximum adsorption capacity of the polymers. *C_e_* (mg/g) was the equilibrium concentration of BPA. *K_L_* (L/mg) was the Langmuir binding constant, which was related to the energy of adsorption. *K_F_*, the Freundlich constant, was related to the adsorption capacity. The constant *n* in the Freundlich equation was an indication of the favorableness of the adsorbent/adsorbate interaction. The Langmuir and Freundlich fits were shown in Figure 5a, and the regression data of the two models for all adsorbents were listed in Table 1. The adsorption of BPA on Mag-PVP and Fe_3_O_4_ nanospheres were more adequately represented by Langmuir than Freundlich. The higher regression coefficient of the Langmuir model (*R*^2^ = 0.94) indicated that the adsorption process for Mag-PVP was a theoretical monolayer adsorption. The maximum adsorption capacity (*Q_m_*) calculated by the Langmuir model for Mag-PVP and Fe_3_O_4_ nanospheres were 115.87 and 42.05 mg/g, respectively. The results suggested that Mag-PVP had a better adsorption capacity than Fe_3_O_4_ nanospheres. The enhancement of adsorption was attributed to the coating of poly-divinylbenzene-2-vinylpyridine of the Fe_3_O_4_ nanospheres, which could provide more interaction sites.

### 3.3. Effect of Contact Time

As shown in Figure 5b, the adsorption amount (*Q_t_*, mg/g) on Mag-PVP increased with an increase in time at the initial stage and nearly reached a plateau after approximately 10 min. The high adsorption rate may be caused by the following factors: the Mag-PVP diameter was very small (approximately 160 nm) and the adsorption property of Mag-PVP was enhanced through the grafting modification of Fe_3_O_4_ nanospheres with DVB and 2-VP. Mag-PVP reached the adsorption capacity of 33.3 mg/g in 10 mg/L of BPA solution. The fast adsorption rate and high adsorption capacity demonstrated that Mag-PVP was an efficient adsorbent for the adsorption of BPA.

### 3.4. Effect of Coexisting Ions and HA

**Figure 6:**
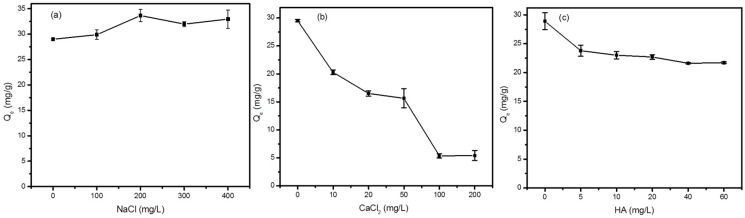
Effect of coexisting Cl^−^ (**a**), Ca^2+^ (**b**), and humic acid (**c**) on BPA adsorption onto Mag-PVP (BPA concentration = 10 mg/L; adsorbent dosage = 0.2 g/L; shaking time = 4 h). Error bars represent standard deviation values from three replicates.

It is well known that environmental water contains not only pollutants, but also high concentrations of salts, which may affect the removal of pollutants [36]. Therefore, the effect of ionic strength on BPA adsorption was investigated by performing adsorption equilibrium experiments at different concentrations of NaCl and CaCl_2_. As shown in Figure 6a, the adsorption capacity of BPA improved in the presence of NaCl. This phenomenon could be explained by the negative charge of Mag-PVP and the molecular form of BPA. The Cl^−^ was placed between the surface of Mag-PVP and the BPA molecules, which produced a screening effect of the surface charge. It favored adsorbate–adsorbent interactions and enhanced the adsorption of BPA. The presence of NaCl in solution also caused a salting-out effect via decreasing the solubility of BPA, thereby enhancing its adsorption onto Mag-PVP. The advantageous effect of electrolytes on BPA adsorption was previously reported for active carbon and graphene [8,13]. The amount of BPA adsorbed decreased rapidly in the presence of CaCl_2_ (Figure 6b). The observed phenomenon was interpreted in terms of the ability of Ca^2+^ to form a calcium–PVP complex, resulting in the adsorption sites of Mag-PVP being occupied by Ca^2+^. Similar findings have been reported in previous studies [37]. Humic acid (HA) is naturally occurring macromolecule in organic matter, which is ubiquitous in the natural environment. It has potential effects on the sorption of chemicals to sorbents [38,39]. The adsorption effect by HA were shown in Figure 6c. When the concentrations of HA were varied from 0–40 mg/L, the adsorption capacity of BPA by Mag-PVP decreased due to the competitive adsorption between HA and BPA on Mag-PVP. The functional groups contained in HA can interact with Mag-PVP, occupying its adsorptive site. When the concentration of HA exceeded 40 mg/L, there was little change in the adsorption capacity, indicating that the adsorptive site of Mag-PVP for adsorbing HA was completely occupied.

### 3.5. Effect of pH

**Figure 7:**
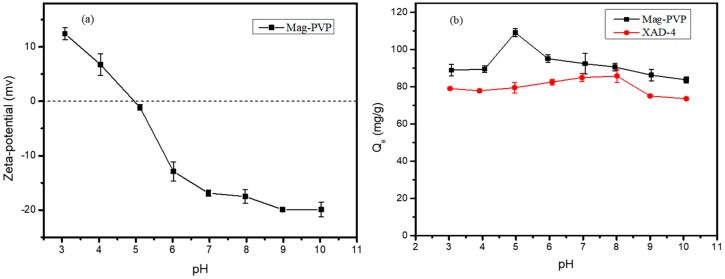
(**a**) Zeta-potential of Mag-PVP as a function of pH values; (**b**) Effect of pH on BPA adsorption by Mag-PVP and XAD-4 (BPA concentration = 20 mg/L; adsorbent dosage = 0.2 g/L; shaking time = 4 h). Error bars represent standard deviation values from three replicates.

BPA is weakly acidic in nature, being ionizable to form organic anions at sufficiently high pH levels. Its behavior in the environment is complex. pH is an important parameter affecting the adsorption process. The zeta-potential of Mag-PVP as a function of pH was shown in Figure 7a. The effect of pH on the adsorption of BPA was shown in Figure 7b. XAD-4, with a cross-linked bridge and benzene ring structure, was selected as the control [15]. Compared to XAD-4, Mag-PVP has N elements in its structure. The adsorption of BPA by Mag-PVP increased slightly from 3.0 to 5.0, and obtained the largest adsorption amount at pH 5.0, which was then reduced with an increase of pH. However, the pH had no significant effect on XAD-4 adsorption capacity until it was higher than 8.0. 

**Figure 8:**
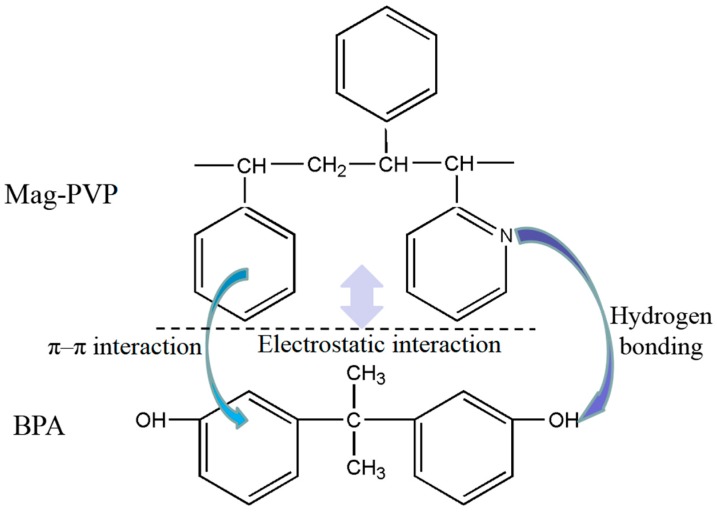
Possible mechanism for BPA adsorption on Mag-PVP.

The point of zero zeta-potential of Mag-PVP was at pH 5.0. Mag-PVP was positively-charged when the pH was less than 5.0. The largest adsorption amount of Mag-PVP at pH 5.0 was probably attributed to hydrogen bonding. The nitrogen of PVP can form hydrogen bonding with the phenol hydroxyl of BPA at pH 5.0. At pH 5.0–9.0, BPA existed in a neutral form, and the nitrogen-containing surface functional groups were deprotonated. The hydrogen bonding interaction decreased, which resulted in the reduced adsorption capacity. BPA is a weak acid with a pKa value of 9.6–10.2 [8]. Its particles release a proton and form bisphenolate anions in alkaline solution. The reduction of BPA adsorption when pH > pKa was obvious because of growing repulsion forces and a reduction of π–π interactions between bisphenolate anions and the surface of the sorbent. The phenomenon was consistent with XAD-4 when the pH was above 8.0. There was no change in the BPA adsorption capacity of XAD-4 at pH < 8.0, indicating that the adsorption mainly depended on the interaction of hydrophobic or π–π interactions, excluding the effect of hydrogen bonding. Above all, three main mechanisms can explain the adsorption of Mag-PVP. Hydrogen bonding, electrostatic interaction and π–π interactions are mutually responsible for the adsorption of BPA onto Mag-PVP. The possible schematic illustration of BPA adsorption onto Mag-PVP was presented in Figure 8. These results also indicate that Mag-PVP could be efficiently applied to weakly acidic and alkaline water for the removal of BPA.

### 3.6. Effect of Environmental Matrix

**Figure 9:**
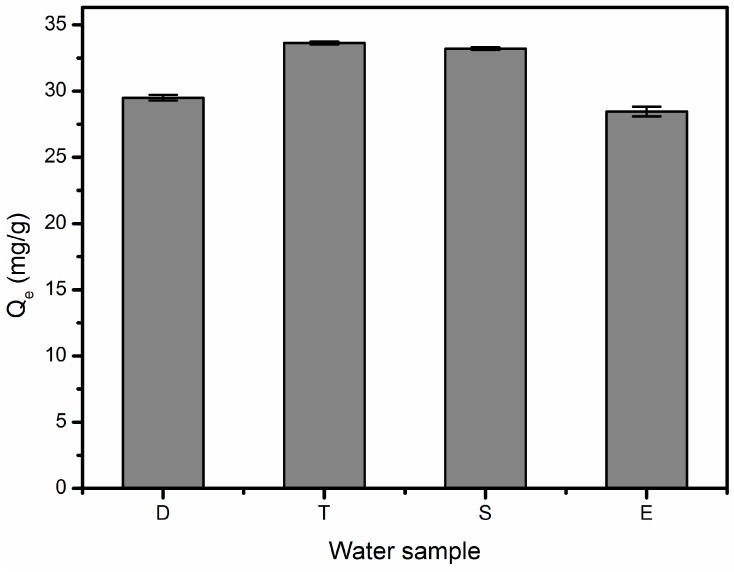
The adsorption of BPA on Mag-PVP in different water samples. D, T, S, and E represent deionized water, tap water, surface water, and the effluent water in sewage treatment plants, respectively (BPA concentration = 10 mg/L; adsorbent dosage = 0.2 g/L; shaking time = 4 h). Error bars represent standard deviation values from three replicates.

The effluent water from Nanjing Jiangxinzhou sewage treatment plants, surface water from the river of Nanjing University Xianlin Campus, deionized water, and a tap water were used to study the effect of sample matrix on BPA adsorption. The main properties of these water samples were shown in Table 2. The adsorption of BPA in tap water and surface water was higher than that obtained in deionized water (Figure 9). The adsorbed amount of BPA in sewage treatment plant water decreased by 4% compared with that in deionized water. All results showed that the effect of environmental matrices on BPA adsorption was not evident. Mag-PVP was an excellent adsorbent to remove BPA from environment water samples.

### 3.7. Regeneration and Reuse

**Figure 10:**
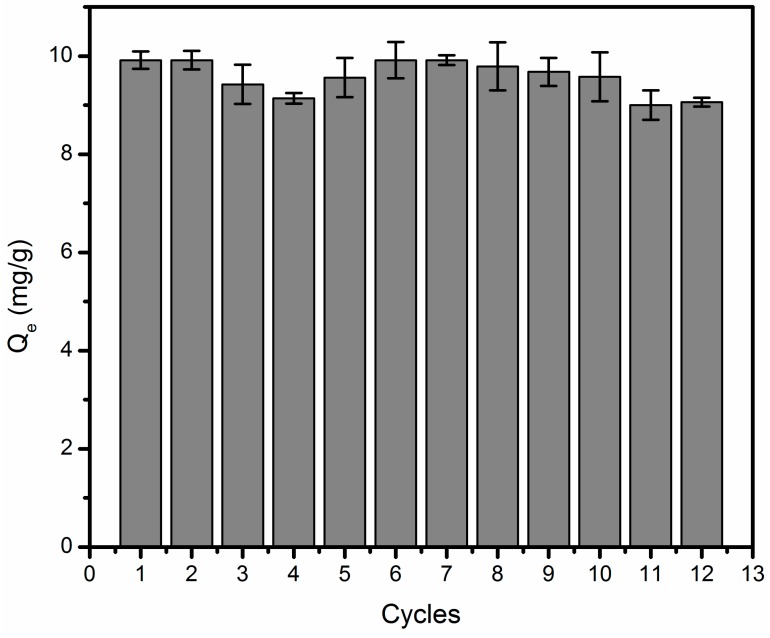
Regeneration performance of Mag-PVP (BPA concentration = 5 mg/L; adsorbent dosage = 0.2 g/L; shaking time = 4 h). Error bars represent standard deviation values from three replicates.

Considering the cost-effective application of Mag-PVP in wastewater treatment, the possibility of regeneration and reusability was further investigated. Desorption of BPA in different organic solvents, such as methanol, ethanol, toluene and cyclohexane, was studied. Results found that methanol had the best desorption efficiency [40]. Methanol was selected for the regeneration of Mag-PVP and the data were shown in Figure 10. Mag-PVP can be recycled at least twelve times without the expense of adsorption capacities. After twelve cycles, the adsorption capacity is around 96%. Excellent reusability performance indicated that Mag-PVP can be used in practical applications for removal of BPA.

### 3.8. Comparison of BPA Adsorption on Mag-PVP with Other Adsorbents

A comparison has been made between Mag-PVP and previously reported adsorbents for BPA adsorption. The maximum adsorption capacity obtained from the Langmuir model was used for the comparison of different sorbents. As shown in Table 3, the maximum adsorption capacity of Mag-PVP (115.9 mg/g) was higher than that of most other sorbent materials. Mag-PVP can be simply synthesized and separated from the mixture solution. Because of these advantages, Mag-PVP can be used in full mixing treatment processes. According to the results above, high adsorption capacity and excellent reusability performance in this work indicated that Mag-PVP was an effective adsorbent for removing BPA from aqueous solutions.

## 4. Conclusions

In this study, a novel 2-vinylpyridine functionalized magnetic nanoparticle (Mag-PVP) was successfully prepared for the decontamination of BPA from aqueous solutions. The prepared Mag-PVP exhibited excellent thermal stability and saturation magnetization, and could be easily separated from the suspension by an external magnetic field. The maximum adsorption capacity (*Q_m_*) of Mag-PVP for BPA obtained from the Langmuir isotherm was 115.87 mg/g at 20 °C, which was more than most other adsorbents. Mag-PVP reached its adsorption equilibrium in approximately 10 min. In the presence of NaCl, the amount of BPA adsorbed at the equilibrium on Mag-PVP improved because of the screening effect of Mag-PVP surface charge and the salting-out effect. In the presence of CaCl2 and HA, the amount of BPA adsorbed at the equilibrium decreased due to competitive adsorption. The adsorption of BPA by Mag-PVP increased slightly with an increase in pH from 3.0 to 5.0 and obtained the largest adsorption amount at pH 5.0, which was attributed to hydrogen bonding interactions in the adsorption process. Mag-PVP showed excellent adsorption performance in removing BPA from environmental water samples. Furthermore, Mag-PVP magnetic composites could be regenerated and reused for adsorption of BPA.

## Figures and Tables

**Table 1:** Parameters of the Langmuir and Freundlich models for the adsorption of BPA on Mag-PVP and Fe_3_O_4_ nanospheres at 20 °C. *K_L_* = Langmuir binding constant. *Q_m_* = maximum adsorption capacity of the polymers. *K_F_* = Freundlich constant. *n* = an indication of the favorableness of the adsorbent/adsorbate interaction.

Temperature (°C)	Langmuir Model			Freundlich Model		
Adsorbents	*K_L_*	*Q_m_*	*R* ^2^	*K_F_*	*n*	*R* ^2^
Mag-PVP	0.09	115.87	0.94	16.20	2.09	0.93
Fe_3_O_4_ nanospheres	0.22	42.05	0.94	11.27	2.67	0.74

**Table 2:** The main properties of the water samples.

Water Samples	Deionized Water (D)	Tap Water (T)	Surface Water (S)	Effluent Water (E)
Temperature (°C)	19.8	19.8	19.9	19.9
pH	7.1	8.3	8.4	8.6
Conductivity (μS/cm)	1.2	241.4	348.3	1120.3
Total organic carbon (mg/L)	0	0.2	2.6	10.1

**Table 3:** Comparison of BPA adsorption on Mag-PVP with other adsorbents.

Name of Sorbent	pH	Temp. (°C)	*Q_m_* (mg/g)	Reference
Mag-PVP	6–7	20	115.9	Our study
Porous carbon produced at 1000 from Moso bamboo	-	23	41.8	[41]
AC purchased from Wako	-	23	56.5	[41]
Modified CNTs	6.0	7	70.0	[42]
Magnetic chitosan fly-ash-cenospheres	7.0	20	31.9	[18]
Magnetic carbon nanotube	6.2	25	45.3	[17]
Multi-walled carbon nanotube	5.2	25	88.27	[43]
Magnetic particles MP_DVB_	8.0	20	90.9	[20]
MAP-GBM	7.0	20	324.0	[44]
Vc-GBM	7.0	25	133.1	[45]
Graphene oxide	6.0	25	87.8	[46]
KOH-activated tyre pyrolysis char	6.5–7.5	25	123.0	[47]

AC = activated carbon. CNT = carbon nanotubes. MP_DVB_ = magnetic polydivinylbenzene. MAP-GBM= magnesium ascorbyl phosphate-graphene-based monoliths. Vc-GBM = three-dimensional-graphene-based monoliths.
